# Studies Regarding As(V) Adsorption from Underground Water by Fe-XAD8-DEHPA Impregnated Resin. Equilibrium Sorption and Fixed-Bed Column Tests

**DOI:** 10.3390/molecules191016082

**Published:** 2014-10-09

**Authors:** Mihaela Ciopec, Adina Negrea, Lavinia Lupa, Corneliu M. Davidesc, Petru Negrea

**Affiliations:** Faculty of Industrial Chemistry and Environmental Engineering Blvd., “Politehnica” University of Timisoara, Vasile Parvan No. 6, Timisoara 300223, Romania

**Keywords:** arsenic removal, Fe-XAD8-DEHPA, underground water, adsorption isotherm, kinetics, column study

## Abstract

The characteristics of arsenic adsorption onto Fe-XAD8-DEHPA resin were studied on the laboratory scale using aqueous solutions and natural underground waters. Amberlite XAD8 resin was impregnated with di(2-ethylhexyl) phosphoric acid (DEHPA) via the dry method of impregnation. Fe(III) ions were loaded onto the impregnated resin by exploiting the high affinity of arsenic towards iron. The studies were conducted by both in contact and continuous modes. Kinetics data revealed that the removal of arsenic by Fe-XAD8-DEHPA resin is a pseudo-second-order reaction. The equilibrium data were modelled with Freundlich Langmuir and Dubinin Radushkevich (D-R) isotherms and it was found that the Freundlich model give the poorest correlation coefficient. The maximum adsorption capacity obtained from the Langmuir isotherm is 22.6 µg As(V)/g of Fe-XAD8-DEHPA resin. The mean free energy of adsorption was found in this study to be 7.2 kJ/mol and the Δ*G°* value negative (−9.2 kJ/mol). This indicates that the sorption process is exothermal, spontaneous and physical in nature. The studied Fe-XAD8-DEHPA resin showed excellent arsenic removal performance by sorption, both from synthetic solution and the natural water sample, and could be regenerated simply by using aqueous NaOH or HCl solutions.

## 1. Introduction

Because in developing countries underground waters represent the main source of drinking water, their contamination with arsenic is a problem which must be solved [1,2,3]. It is well known that because of its toxicity the presence of arsenic in drinking water above the maximum admitted level has a negative impact on human health [1,2,3,4,5,6,7,8]. In order to reduce its adverse health effects it is necessary to minimize the pollution of underground water with arsenic content or to find some effective methods for their remediation [3]. The valences and inorganic species of arsenic depend on the redox conditions and the pH of the underground waters. In an aqueous solution of As(V) there are four species: H_3_AsO_4_, H_2_AsO_4_^−^, HAsO_4_^2−^, AsO_4_^3−^ that predominate at pH values between 6 and 9. In the same mode in aqueous solutions at pH values below 9 they exist the As(III) species: H_3_AsO_3_, H_2_AsO_3_^−^, HAsO_3_^2−^, AsO_3_ [4,5,6,9,10]. The conventional methods used for the arsenic removal from aqueous solutions are precipitation, coagulation and filtration, reverse osmosis, ion exchange and adsorption. Of the above methods, adsorption is the most promising one as it is economical and highly efficient [1,2,3,4,5,6,7,8,9,10,11,12]. In the last years solvent impregnated resins (SIR) were used for the recovery and selective separation of metal ions from aqueous solutions with good results [13,14,15,16,17,18,19,20,21,22]. In order to improve the adsorption properties of the SIRs four method of impregnation with an organic extractant of the polymeric support were developed. These are: the dry, wet, modifier addition and dynamic column methods [13,16,22,23,24]. In the present study, the dry method of impregnation was used to impregnate an Amberlite XAD8 resin with di(2-ethylhexyl) phosphoric acid DEHPA in order to obtain an efficient adsorbent for the removal of As(V) from aqueous solutions [22]. We focused on this resin because in our previous studies the Amberlite XAD7 resin displayed good adsorption properties in the process of arsenic removal from aqueous solutions [18]. The advantages of the use as solid support of the Amberlite XAD series are represented by the possibilities of obtaining spherical beads of suitable size. Other researchers used these resins for the arsenic removal, but in other more expensive functionalization systems [20,21]. Due to the shape of these solid supports they are easy to separate from the solutions and also easy to regenerate, avoiding in this way the drawbacks of the adsorption method. On the other hand the purpose of our research was to obtain an adsorbent which could be efficiently used in the process of arsenic removal from aqueous solutions having concentrations under 100 μg/L. These concentrations were the most common found in the underground waters from the west area of Romania and east area of Hungary [25,26]. Awual and co-workers underlined the importance of the preparation of an adsorbent intended to remove arsenic from waters containing low concentrations. They also demonstrated that the metal-loaded ligand exchangers show rapid, selective and great efficiency in the process of trace arsenic removal from aqueous solutions [27,28,29,30,31]. For this reason, in this paper, in order to prepare an efficient adsorbent for the purification of water with trace levels of arsenic, the XAD8-DEHPA impregnated resin was loaded with Fe(III) ions. We used the iron ions for the metal loading of the impregnated resin due to the high affinity of arsenic towards iron [3,4,7,8,9,10,11,24,32]. By using as adsorbent material, in the process of arsenic removal from aqueous solutions, a resin loaded with a polyvalent metal ion, combines the known advantages of the resin such as excellent hydraulic properties and mechanic strength along with the excellent selectivity offered by the loaded metal ion [33]. Even if the metal ion content (weight %) of the loaded resin is much less than the metal oxide, the resulting adsorbent presents a higher efficiency due to its mechanical integrity and its possibility to be used for several cycles [34]. Taking into account these considerations the purposed adsorbent material is in agreement with the Life Cycle Approach, underlining the sustainability of arsenic removal through adsorption [35]. The objective of this study was to investigate the adsorption characteristic of the Fe-XAD8-DEHPA resin for As(V) removal in aqueous solutions by performing equilibrium sorption tests and fixed-bed column tests.

## 2. Results and Discussion

### 2.1. Evaluation of the Adsorbent Preparation Process

**Figure 1:**
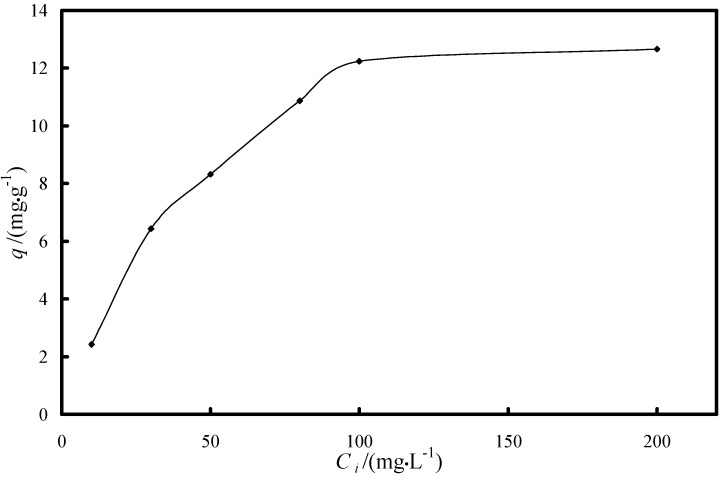
Dependence of the Fe(III) ions uptake versus the initial concentration of Fe(III) pH = 3, *t* = 24 h, *m* = 0.1 g, *V* = 25 mL.

The extractant content of the XAD8-DEHPA was found to be 0.28 g DEHPA for each gram of SIR as determined by titration using 0.1 M NaOH. The experimental data regarding the dependence of the Fe(III) ion uptake versus the initial concentration on Fe(III) ions in the loading process of iron onto XAD8-DEHPA resin are presented in Figure 1.

The experimental data shows the increase of the Fe(III) ions uptake as the initial concentration of Fe(III) ions in solution increases. Above 100 mg/L initial Fe(III) concentration, there is no significant increase of the Fe(III) ions loaded onto the XAD8-DEHPA. Therefore it was considered that for the loading of the XAD8-DEHPA resin, using a S:L ratio of 0.1:25, the concentration of the Fe(III) ions in the aqueous solutions should not exceed 100 mg/L. In this way the loading of the highest quantity of iron ions onto the impregnated resin is obtained, which will lead to a higher adsorption of As(V) ions from the aqueous solution, due to the fact that the Fe(III) ions are responsible for the arsenic removal because of its affinity for this pollutant [3,4,7,8,9,10,11,24,32].

**Figure 2:**
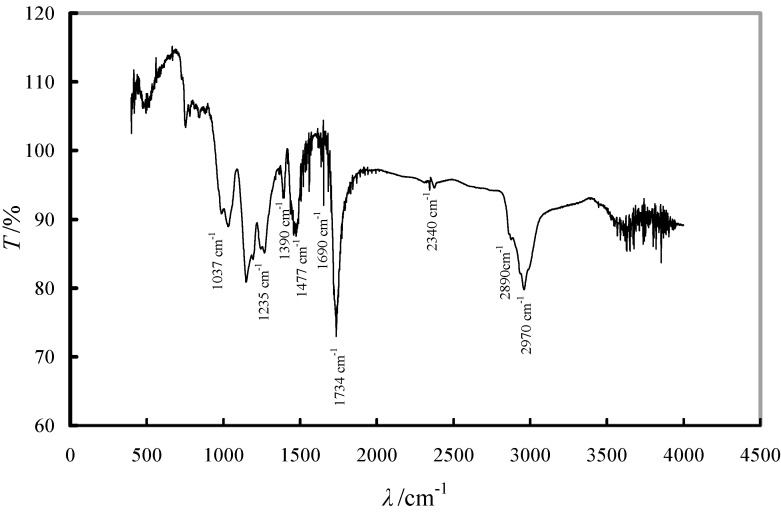
IR spectrum of a Fe-XAD8-DEHPA sample.

The IR spectrum of Fe-XAD8-DEHPA (Figure 2) presents the characteristic bands of XAD8 resin and also illustrated the fact that the resin was impregnated with the studied solvent and loaded with the Fe(III) ions. The peak at 1390 cm^−1^ was assigned to the C-H deformation vibration of CH_3_; the absorption band at 1734 cm^−1^ corresponds to a C=O stretching vibration and the band at 2970 cm^−1^ was attributed to the C-H stretching vibration of CH_3_. The impregnated status of the XAD8 resin by DEHPA extractant is manifested in the appearance of the adsorption band at 1235 cm^−1^, which is the P=O stretching band. P-CH_2_ stretching is observed at 1477 cm^−1^; the O-H bending band was found at 1690 cm^−1^ and the band assigned to P-OH is observed at 2340 cm^−1^. The iron loading of the XAD8-DEHPA resin is demonstrated by the peaks at 1037 and 2890 cm^−1^ which belong to the Fe-OH stretching band.

**Figure 3:**
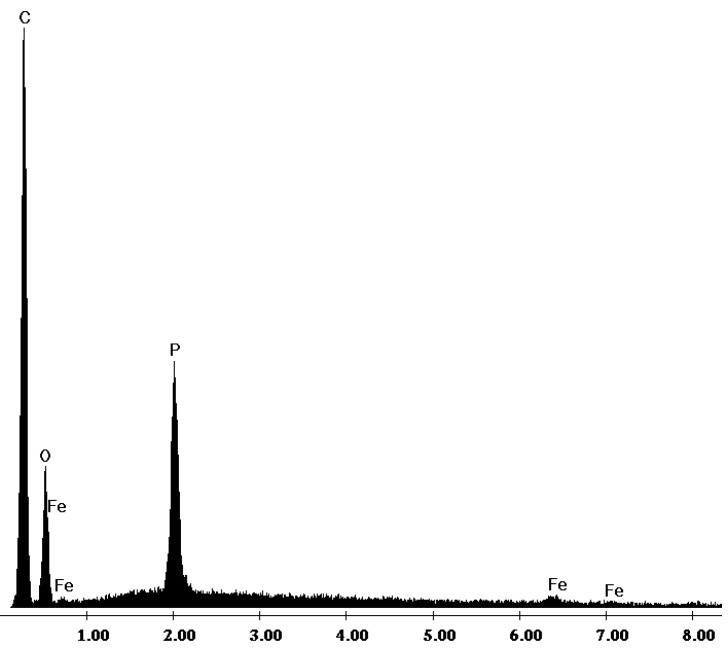
EDX spectrum of Fe-XAD8-DEHPA sample.

The impregnation of the resin with DEHPA and the loading of the impregnated resin with Fe(III) ions is also demonstrated by the EDX spectra, where characteristic peaks of phosphorous ions arising from the DEHPA solvent and iron were identified (Figure 3).

### 2.2. Sorption Equilibrium Tests

#### 2.2.1. Study of the Kinetics of Adsorption

**Figure 4:**
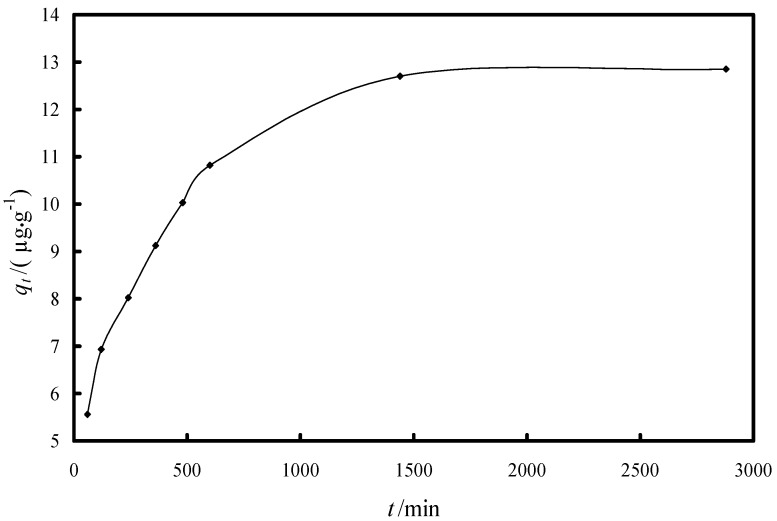
Effect of contact time on the As(V) adsorption capacity of Fe-XAD8-DEHPA pH = 9 ± 0.1; *T* = 298 ± 1 K; *C*_0_ = 100 µg/L; *m* = 0.1 ± 0.0001 g; *V* = 25 ± 0.1 mL.

In order to determine the time when the equilibrium between the arsenate and the impregnated resin is reached kinetic studies were performed. The effect of contact time on the equilibrium adsorption capacity of Fe-XAD8-DEHPA during the removal process of As(V) from aqueous solution at room temperature is presented in Figure 4. According to this data, saturation was reached after 24 h contact time. At an initial 100 ppb As(V) concentration value, the equilibrium adsorption capacity was determined to be ~13 μg As(V)/g of impregnated resin.

**Figure 5:**
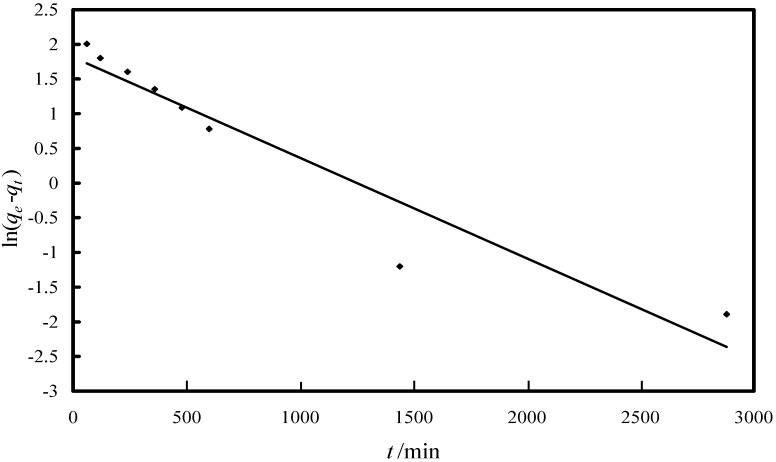
Linear model of the pseudo-first-order reaction kinetics of As(V) adsorption onto Fe-XAD8-DEHPA resin.

In order to investigate the arsenic adsorption mechanism onto the Fe-XAD8-DEHPA resin, three kinetic models, namely (i) pseudo-first-order equation of Lagergren based on solid capacity; (ii) pseudo-second-order reaction model of Ho and Mckay based on the solid phase sorption; and (iii) intra-particle diffusion model were tested. Descriptions of the integral form of the models are given in the following. The pseudo-first-order kinetic model is defined by the Equation [14,36]:

ln(*q_e_* – *q_t_*) = ln *q_t_* – *k*_1_·*t* (1)

where *q_e_* and *q_t_* are the amount of the As(V) adsorbed onto the Fe-XAD8-DEHPA (µg/g) at equilibrium and after time *t*, respectively. *t* is the contact time (min), *k*_1_ is the specific sorption rate constant (min^−1^). The values of the adsorption rate constant (*k*_1_) were determined from the ln(*q_e_ − q_t_*) in terms of *t* (Figure 5).

**Figure 6:**
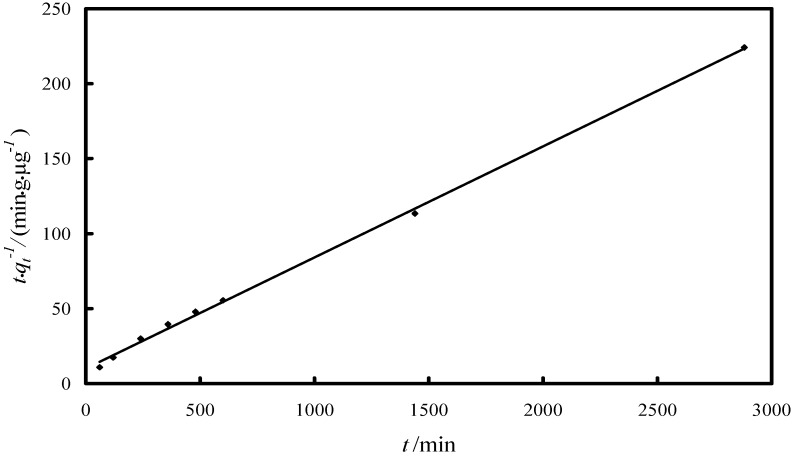
Linear model of the pseudo-second-order reaction kinetics of As(V) adsorption onto Fe-XAD8-DEHPA resin.

The linear form of the pseudo-second order model is defined by [2,37]:

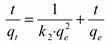
(2)
where *q_e_* and *q_t_* are the amount of the As(V) adsorbed onto the studied material (µg·g^−1^) at equilibrium and at time *t*, respectively. *t* is the contact time (min), *k*_2_ is the pseudo-second-order adsorption rate constant (g·µg^−1^·min^−1^). The value *q_e_* and *k*_2_ are determined from the slope and intercept of (*t·q_t_*^−1^) *versus t* (Figure 6). In Equation (2), the expression *k*_2_·*q_e_*^2^ in the intercept term describes the initial sorption rate *h*/(µg·(g·min)^−1^) as t → 0.

**Figure 7:**
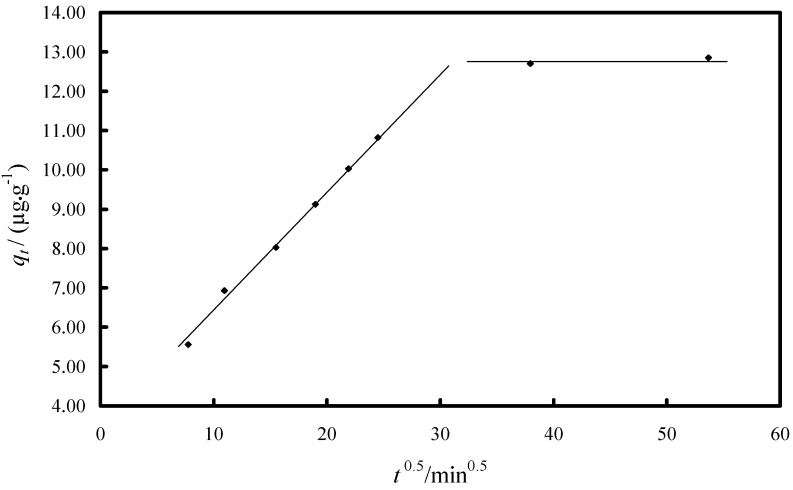
The Weber-Morris plot for intra-particle diffusion of the As(V) adsorption kinetic data on Fe-XAD8-DEHPA resin.

Intra-particle diffusion is an important phenomenon for sorption processes in porous materials. The adsorption of As(V) ions onto the Fe-XAD8-DEHPA may be controlled via external film diffusion at earlier stages and later by the particle diffusion. The possibility of intra-particle diffusion resistance was identified by using the following Weber-Morris intra-particle diffusion model [6,7,9]:

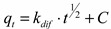
(3)
where *k_dif_* is the intra-particle diffusion rate constant (µg·g^−1^·min^−1/2^) and *C* is the intercept. The values of *q_t_* versus *t*^1/2^ and the rate constant *k_dif_* are directly evaluated from the slope of the regression line (Figure 7).

The application of the different kinetic models unveiled some interesting features regarding the mechanism and rate-controlling step in the overall sorption process. The values of the constants, together with the regression coefficients (R^2^) obtained in all cases are summarized in Table 1.

A poor regression of the pseudo-first-order equation infers that this model does not adequately describe the present adsorption process. Further, there is a great difference between the *q_e_* values obtained experimentally and the values obtained directly from the kinetic plot (Figure 5). At the same time, the correlation coefficient for the linear plot of the pseudo-second-order kinetic plot is excellent, greater than 0.99. The *q_e_* values experimentally obtained shows excellent agreement with the value calculated from the kinetic plot. This shows that the kinetic of As(V) removal by Fe-XAD8-DEHPA can be well described by a pseudo-second-order expression.

**Table 1:** Kinetic parameters for As(V) adsorption onto Fe-XAD8-DEHPA resin.

Model/Parameter	Value
*Pseudo-first-order model*	
*k*_1_/min^−1^	0.0014
R^2^	0.9158
*q*_e_ (experimental)/(µg·g^−1^)	13
q_e_ (kinetic plot)/(µg·g^−1^)	6.13
*Pseudo-second-order model*	
k_2_/(g·µg^−1^·min^−1^)	5.45 × 10^−4^
R^2^	0.9957
*q*_e_ (experimental)/(µg·g^−1^)	13
*q*_e_ (kinetic plot)/(µg·g^−1^)	13.5
*Intra-particle diffusion model*	
*k*_dif_/(µg·g^−1^·min^−1/2^)	0.3049

In order to obtain some information regarding which is the rate-limiting step (boundary layer diffusion or intra-particle (pore) diffusion of solute towards the solid surface) of the arsenic adsorption process onto Fe-XAD8-DEHPA resin, the Weber-Morris intraparticle diffusion model was studied. The straight line which passes through origin obtained from the plot of *q_t_* against square root of time shows that the rate limiting step is the intraparticle diffusion [38,39]. The deviation of the plot from the line indicates the rate-limiting step should be boundary layer (film) diffusion controlled. The plot of *q_t_ versus t*^0.5^ in Figure 7 shows that the dependence is not linear for the duration of entire reaction. The line does not pass through the origin, and thus, intra particle diffusion cannot be the rate-limiting step in the sorption process. The plot shows two straight sections, suggesting two different types of sorption mechanisms at the beginning and at the end of the process. The first linear section indicates initial rapid uptake due to film diffusion and consequent external surface coverage by the sorbate. The second linear section suggests the transportation of sorbate inside the sorbent particles [38,39]. This suggests that the As(V) removal by Fe-XAD8-DEHPA is a complex process.

#### 2.2.2. Study of the Adsorption Isotherm

**Figure 8:**
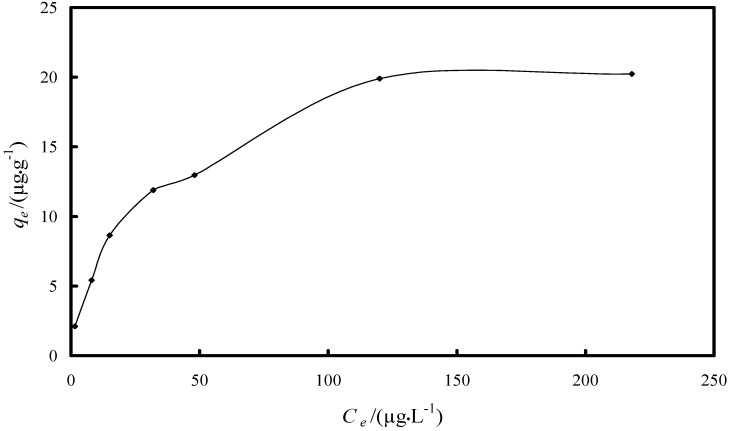
Adsorption isotherm of As(V) ions onto Fe-XAD8-DEHPA resins; *T* = 298 ± 1 K; pH = 9 ± 0.1; *C*_0_ = range:10 to 300 μg·L^−1^; *m* = 0.1 ± 0.0001 g; *V* = 25 ± 0.1 mL; *t* = 24 h.

The adsorption isotherm of As(V) is presented in Figure 8. At high equilibrium concentrations, the adsorption capacity approaches a constant value. This value represents the experimentally determined maximum adsorption capacity of As(V) onto Fe-XAD8-DEHPA (*q_m exp_* > 20.2 μg·g^−1^).

**Figure 9:**
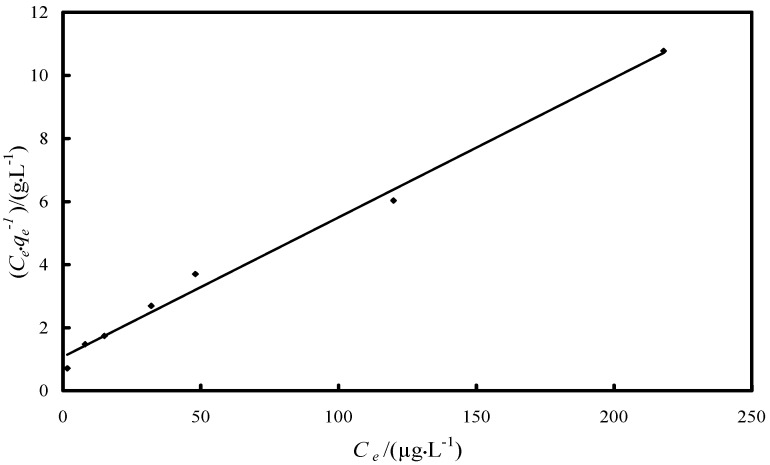
Langmuir isotherm model for As(V) adsorption onto Fe-XAD8-DEHPA resin.

**Figure 10:**
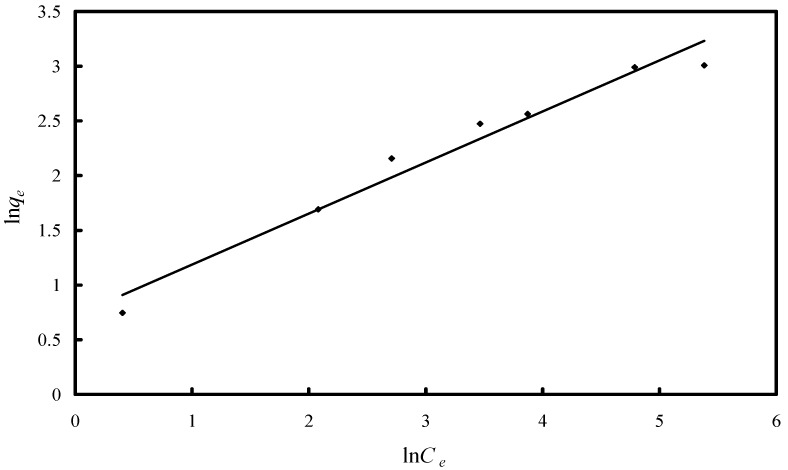
Freundlich isotherm model for As(V) adsorption onto Fe-XAD8-DEHPA resin.

Langmuir and Freundlich isotherm studies were conducted in order to investigate the maximum adsorption capacity of Fe-XAD8-DEHPA resin towards As(V) [40]. The relation between the amounts of adsorbate adsorbed by the adsorbent can be expressed by the linearized Langmuir adsorption isotherm as:

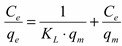
(4)
and the Freundlich isotherm as:

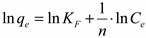
(5)
where *q_e_* is the amount of adsorbate adsorbed per unit of the adsorbent (µg·g^−1^), *K_L_* the adsorption constant related to the enthalpy of adsorption (L·μg^−1^), *C_e_* the equilibrium concentration of As(V) (µg·L^−1^), *q_m_* the maximum adsorption capacity (µg·g^−1^), and *n* and *K_F_* are the constants depending upon the nature of the adsorbate and adsorbent where *n* represents the adsorption intensity and *K_F_* represents the adsorption capacity (µg·g^−1^). A linear Langmuir isotherm was drawn by plotting *C_e_·q_e_*^−1^ *versus C_e_* (Figure 9), while Freundlich isotherm for the adsorption was drawn by plotting ln*q_e_ versus* ln*C_e_* (Figure 10). The calculated parameters, as well as the correlation coefficients (R^2^) for As(V) removal through adsorption onto Fe-XAD8-DEHPA are presented in Table 2*.*

**Table 2:** Parameters of Freundlich, Langmuir and D-R isotherms for As(V) adsorption onto Fe-XAD8-DEHPA resin.

Isotherm/Parameter	Value
*Langmuir isotherm*	
*K_L_/(*L·µg^−1^)	0.041
*q_m_*/(µg·g^−1^)	22.6
R^2^	0.9918
*Freundlich isotherm*	
*K_F_*/(µg·g^−1^)	2.055
*1/n*	0.4664
R^2^	0.9667
*D-R isotherm*	
*k*/(mol^2^·kJ^−2^)	0.0096
*q_m_*/(µg·g^−1^)	24.16
R^2^	0.9921

The Freundlich plot has a lower correlation coefficient than the Langmuir plot. This suggests that the use of the Freundlich isotherm is limited. The Langmuir model effectively describes the equilibrium sorption data (Figure 9); the linear plot has a good correlation coefficient (>0.99) and the maximum adsorption capacity is close to that determined experimentally. The isotherm describes the sorption process for the entire studied concentration range. The maximum adsorption capacity, developed by the studied adsorbent in the removal process of arsenic from water, was compared with other adsorption capacities developed by other materials loaded with iron ions presented in literature (Table 3).

**Table 3:** Maximum adsorption capacities develop by various adsorbent loaded with iron ions in the removal process of arsenic from water.

Adsorbent	*q_m_*, µg·g^−1^	References
Fe-XAD8-DEHPA	22.6	Present work
Fe-XAD7-DEHPA	17.6	[18]
Fe-IR-120(Na)-DEHPA	21.8	[41]
Alginate bead (doped and coated with iron ions	14	[42]
Iron oxide coated sand	8	[43]
Iron oxide coated sand-2	18	[44]

It can be notice that the present adsorbent developed a higher efficiency in the removal process of As(V) from aqueous solutions compared with other iron loaded materials present in the literature. The studied adsorbent can be used with success for arsenic removal from waters containing trace concentrations, the most often situation for drinking waters.

However, neither Langmuir nor Freundlich isotherms provide information about the adsorption mechanism. In order to assess the type of adsorption process, the Dubinin-Radushkevich (D-R) isotherm was also employed [45]. This can be expressed as:

ln*q_e_* = ln *q_m_* – *k*·*ε*^2^ (6)

where ε (Polanyi potential) is given as:

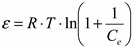
(7)

**Figure 11:**
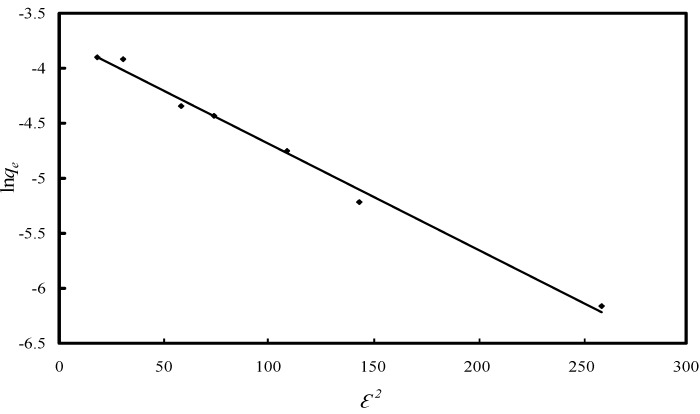
D-R isotherm model for As(V) adsorption onto Fe-XAD8-DEHPA resin.

In the above expression, *q_e_* is the amount of As(V) adsorbed (mg·g^−1^) at equilibrium per unit weight of adsorbent, *q_m_* the maximum adsorption capacity (mg·g^−1^), *C_e_* the equilibrium concentration of As(V) in the solution (ppm), *k* the constant related to adsorption energy (mol^2^·kJ^−2^), R the universal gas constant (kJ·mol^−1^·K^−1^) and *T* is the temperature (K). The D-R isotherm was drawn by plotting ln*q_e_* against ε^2^ (Figure 11). *q_m_* and *k* where calculated from the slop and intercept of the graph and the obtained values are presented in Table 2.

The D-R plot has a good correlation coefficient (>0.99). The monomolecular adsorption capacity *q_m_* for Fe-XAD8-DEHPA in the removal process of As(V) from aqueous solutions is 24.16 µg·g^−1^; a value which is very close to that determined experimentally. The mean free energy of adsorption (*E*), defined as free energy change when one mole of ion is transferred from infinity in solution to the surface of the solid, was calculated from the *k* value using the following formula [22,45]:

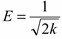
(8)

The magnitude of *E* is a indicator which shows if the adsorption is physical in nature (*E* ≤ 8 kJ·mol^−1^) or chemical (*E* is in the range of 8–16 kJ·mol^−1^) [22,40]. The value of *E* found in this study was 7.2 kJ·mol^−1^, suggesting a physical adsorption (due to weak van der Waals forces).

In order to predict the adsorption efficiency of the adsorption process and to assess if the process is favourable or unfavourable for the Langmuir type adsorption, the isotherm shape can be classified by a term *R_L_*, a dimensionless constant separation factor, which is defined by the following equation
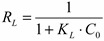
(9)
where *C*_0_ is the initial concentration of As(V) (ppb) and *K_L_* is the Langmuir isotherm constant. The value of *R_L_* indicates the shape of the isotherm to be unfavourable, *R_L_* >1; linear, *R_L_* = 1; favourable 0 < *R_L_* < 1; and irreversible, *R_L_* = 0 [5,6,7,12,36]. In our case *R_L_* values were found to be between 0 and 1 for all the concentration of As(V) ions showing that the adsorption is favourable. Furthermore, the standard Gibbs free energy changes (Δ*G°*) for the adsorption process can be calculated by using the following equation:
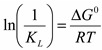
(10)
where *K_L_* is the Langmuir isotherm constant (L·mg^−1^), *R* the universal gas constant (8.314 J·mol^−1^·K^−1^), and *T* is the absolute temperature (*K*). The calculated Δ*G*° value is −9.2 kJ·mol^−1^. The negative Δ*G*° value indicates that the adsorption is spontaneous.

### 2.3. Fixed-Bed Column Study

The efficiency of the treatment technique depends on the concentration and species of arsenic as well as on the presence of other constituents in the water. A fixed bed column study was conducted with real arsenic bearing ground water. The real arsenic-bearing water sample had the composition: NO^3−^: 20 mg·L^−1^; NO^2−^: 0.5 mg·L^−1^; P_2_O_5_: 5 mg·L^−1^; SO_4_^2−^:10.2 mg·L^−1^; NH_4_^+^: 6.4 mg·L^−1^; Fe^n+^: 1.8 mg·L^−1^; Mn^2+^: 0.6 mg·L^−1^; Na^+^: 120 mg·L^−1^; K^+^: 1.75 mg·L^−1^; Ca^2+^: 30 mg·L^−1^; Mg^2+^: 18 mg·L^−1^; As^n+^: 80 μg·L^−1^.

**Figure 12:**
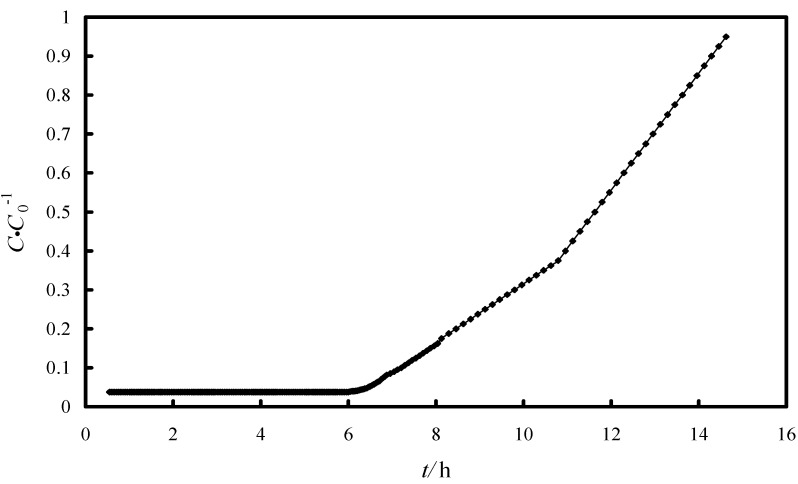
Breakthrough curve for arsenic using Fe-XAD8-DEHPA.

**Figure 13:**
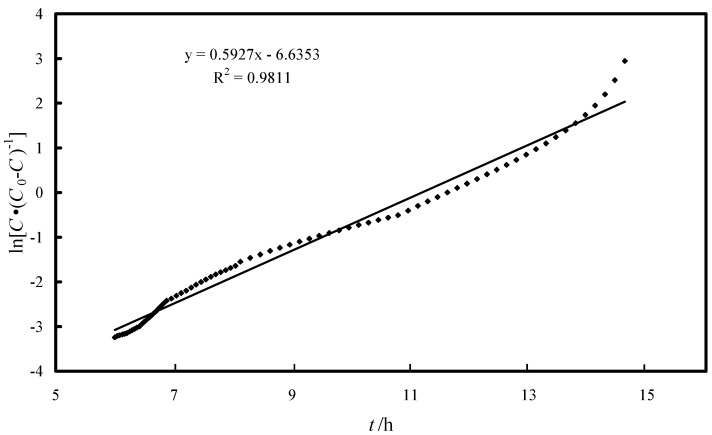
Linearized form of logit model.

The breakthrough curve is shown in Figure 12. The breakthrough time obtained was 6 h and corresponds to *C/C*_0_ = 0.0375 (when 3 L of arsenic-bearing water were treated), while the exhaust time, that corresponds to *C/C*_0_ = 0.5 was found to be at 14.6 h (when 7.3 L arsenic-bearing water were treated). The fixed bed column was designed by the logit method [45,46]. The logit equation can be written as:

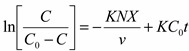
(11)
where *C* is the solute concentration at any time *t*, *C*_0_ the initial solute concentration (80 ppb), *v* the approach velocity (~7.6 cm/h), *X* the bed depth (5 cm), *K* the adsorption rate constant (L·(μg·h)^−1^), and *N* is the adsorption capacity coefficient (μg·L^−1^). Plot of ln[*C*·(*C_0_* − *C*)] versus t is shown in Figure 13. The value of adsorption rate coefficient (*K*) and adsorption capacity coefficient (*N*) was obtained as 7.41/(L·(mg·h)^−1^) and 10.09/(mg·L^−1^). These values could be used to design the adsorption column.

### 2.4. Quality Parameters of the Effluent Water

The quality parameters of the effluent water after column adsorption were also determined. It was observed that beside arsenic, the studied adsorbent could also remove iron and manganese ions from the sample (residual concentrations fell under the AAS limits of detection of 0.01 mg·L^−1^ for iron and manganese and 0.01 µg·L^−1^ for arsenic). These results are in agreement with other studies where it was observed that the arsenic was quantitatively removed together with iron and manganese ions [47]. The pH of the effluent water remained almost the same as in the influent water suggesting that no post treatment is necessary. All these indicate that the Fe-XAD8-DEHPA resin could be used as an efficient and practical adsorbent for arsenic removal from underground waters.

### 2.5. Desorption Study

From the standpoints of cost-effectiveness and environmental friendliness; it is highly desirable that the sorbent to be amenable to efficient regeneration and re-use. We found that the regeneration capacity of exhausted Fe-XAD8-DEHPA increased with the increasing concentration of the used regenerant (HCl or NaOH). The adsorbent could be efficiently regenerated by using a 5% HCl solution, but in this case Fe ions loaded onto XAD8-DEHPA resin leached out together with the arsenic ions. Consequently the regenerated resin should in such cases be reloaded with Fe(III) ions before re-use, which somewhat increases the cost of the adsorption process. For this reason, we consider the 5% NaCl solution to be the better regenerant, with the use of which the desorption performance is still 92%.

Even if some authors mentioned that the use of ion-exchange for arsenic removal present a better Environmental Sustainability Assessment compared with the adsorption process [48], the research from this paper showed that the use of Fe-XAD8-DEHPA as adsorbent material in the As(V) removal process presents good efficiency both in batch and fixed-bed column tests, and especially in the treatment process of real underground drinking water, without the applying any extra pre-treatment processes. The adsorbent material can be regenerated and efficiently reused in the adsorption process in this way decreasing the treatment costs. All these results underline the sustainability of arsenic removal through adsorption.

## 3. Experimental Section

### 3.1. Adsorbent Preparation

**Scheme 1:**
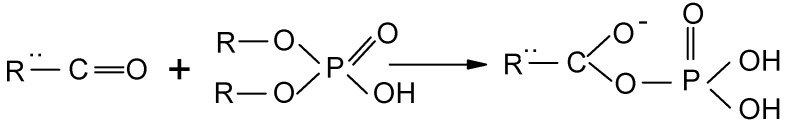
The mechanism of XAD8 impregnation with DEHPA.

The Amberlite XAD8 resin (Rohm and Haas Co., Philadelphia, PA, U.S.A.) was impregnated with di(2-ethylhexyl) phosphoric acid (DEHPA) via the dry method. This macro reticular polymeric adsorbent is dimensionally and chemically quite stable and quite insoluble. The impregnation procedure was described elsewhere [18,22]. Scheme 1 shows the possible mechanism of impregnation.

**Scheme 2:**
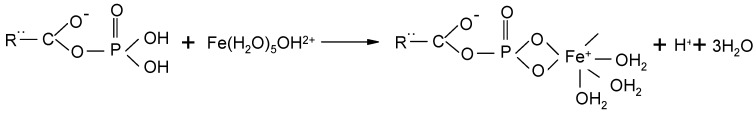
Loading mechanism of Fe(III) ions onto XAD8-DEHPA resin.

The resulted phosphorylated resin was further loaded with Fe(III) ions. The hydroxyl iron ions after coordination in the aqueous medium with the neutral waters molecules are loaded onto the impregnated resin according to the reaction mechanism described in Scheme 2.

In order to determine the maximum quantity of Fe(III) ions that can be loaded onto the impregnated resin, the influence of the Fe(III) ions initial concentration onto the Fe(III) uptake was investigated. To this end 0.1 g of resin was suspended in 25 mL of Fe(III) ions solutions (Fe(NO_3_)_3_ Merck Standard solution) each having a different Fe(III) concentrations in the range from 10 to 200 mg·L^−1^). The suspensions were let to stand in contact for 24 h in order to reach the equilibrium. After this contact time the samples were filtrated and the concentration of iron in the clear solutions was determined using a Varian SpectrAA 280 type atomic absorption spectrometer (Varian Australia Pty Ltd., Mulgrave, VIC, Australia) equipped with an air-acetylene flame atomizer. The optimum concentration of the Fe(III) ions solution for the XAD8-DEHPA resin loading was found to be 100 mg·L^−1^. A higher quantity of XAD8-DEHPA impregnated resin was equilibrated with a 100 mg·L^−1^ Fe(III) ions solution in the S:L ratio S:L = 0.1:25 for 24 h. Fe loaded XAD8-DEHPA resin was separated by vacuum filtration, washed with distilled water until pH was neutral and dried at 323 K. The obtained Fe-XAD8-DEHPA was submitted to FTIR spectroscopy and EDX analysis in order to establish if the impregnation with DEHPA and the iron loading occurred. The FTIR spectra of KBr-pelletized samples were recorded using a (Prestige-21) FTIR spectrophotometer (Shimadzu Europa GmbH, Duisburg, Germany) in the range of 4000–400 cm^−1^ with 2 cm^−1^ resolution and 40 scans were performed. The EDX study was performed using an Inspect S50 scanning electron microscope (FEI, Hillsboro, OR, USA).

### 3.2. Batch Experiments for Arsenic Removal

**Scheme 3:**
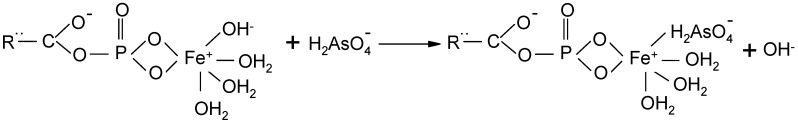
The mechanism of arsenate adsorption onto Fe-XAD8-DEHPA resin.

Due to the fact that in aqueous solution at pH value of 9 ± 0.1 the anionic species of As(V) [H_2_AsO_4_^−^ or HAsO_4_^2−^] are the predominant ones [6,9,11], all the experiments were carried out at this pH value. The pH of the solutions was adjusted to this value using a 1 M NaOH solution, thereby keeping the volume variation of the solution to a value as low as possible. The pH was controlled with MultiMeter MM41 (Crison, Barcelona, Spain) fitted with a glass electrode calibrated by using various buffer solutions. Prior to the equilibrium sorption experiments, a stock solution of arsenic in 0.5 M HNO_3_ was prepared using an appropriate amount of H_3_AsO_4_ (Merck, Darmstadt, Germany). Other solutions of As(V) ions were prepared from the stock solution by appropriate dilution. The arsenate adsorption onto the Fe(III) loaded XAD8-DEHPA resin is mainly attributable to the adsorption of monovalent arsenate anions H_2_AsO_4_^−^. The adsorption of monovalent arsenate may take place by releasing hydroxyl anions or neutral water molecules from the coordination sphere of the Fe(III) ions loaded onto the XAD8-DEHPA resin (Scheme 3), similar to the mechanism proposed for other iron or zirconium-based adsorbents [28,31,32,35,36,37,38,39,40,41,42,43,44,45,46,49,50,51].

In the first experiment the effect of contact time was studied. The arsenic adsorption performance of the Fe-XAD8-DEHPA the experiments was assessed using 0.1 g of the adsorbent and 25 mL of a 100 µg·L^−1^ As(V) solution. The samples were kept in contact for different times (range: 1–24 h) at the room temperature 298 K. After the contact time, the suspensions were filtered and the residual arsenic concentration in the filtrates was determined by hydride generation atomic absorption spectrometry, using a Varian SpectrAA 110 flame atomic absorption spectrometer equipped with a Varian VGA 77 hydride generation system. Similar equilibrium experiments were performed to study the influence of the initial As(V) concentration (10, 30, 50, 80, 100, 200 and 300 µg·L^−1^). In each experiment, 0.1 g of studied material was suspended in 25 mL of As(V) solution of different concentration (range: 10–300 µg·L^−1^) for 24 h at the room temperature. After the contact time the filtrate was collected and subjected to arsenic analysis.

The adsorption capacity of the impregnated resin expressed as the metal uptake, *q*/µg·g^−1^, can be obtained using the next mass balance expression [36,45,51]:

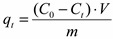
(12)
where *C*_0_ and *C_t_* are the concentrations of As(V) ions (μg·L^−1^) in the solution initially (*t* = 0) and after a time *t* (min), respectively, *V* is the volume of the solution and *m* is the mass of adsorbent. The experimental results are given as an average of five sets of data obtained under identical working conditions.

### 3.3. Fixed-Bed Column Study for Arsenic Removal

The arsenic adsorption in dynamic regime was made on a fixed-bed column packed with Fe-XAD8-DEHPA resin. The installation was composed from: a glass column with an i.d. of 2 cm and 30 cm length, where the impregnated resin layer was of 5 cm, a vessel which contain the arsenic-containing water from where it was pumped with the help of a peristaltic pump in the column in the down-flow mode with a volumetric flow rate of 8.33 mL·min^−1^ (0.796 m^3^·m^−2^·h^−1^). At the bottom of the column the samples were collected at certain time intervals and were analyzed for their arsenic concentrations.

### 3.4. Desorption Studies

Desorption of arsenic from the exhausted Fe-XAD8-DEHPA resin was carried out using 1 g of exhausted adsorbent, 25 mL of HCl solution and NaCl solutions of various concentrations (1%, 3%, and 5%) by magnetic agitation for 2 h at the room temperatures. After the contact time the filtrate was collected for arsenic analysis.

All chemicals employed in the experiments were A.R. grade and used without further purification. Distilled water was used throughout.

## 4. Conclusions

In the present study we have shown that Fe-XAD8-DEHPA adsorbent can be efficiently used for the removal of arsenic from natural underground waters. The adsorption mechanism appears to follows a pseudo-second-order kinetics. The presence of intraparticle diffusion showed that adsorption process is complex and involves multiple mechanisms. The adsorption was also evaluated according to the Langmuir, Freundlich and D-R isotherm models, obtaining a maximum adsorption capacity from the Langmuir isotherm of 22.6 µg As(V)/g of Fe-XAD8-DEHPA resin. The values obtained from the fixed-bed column test could be used to design an adsorption column. In addition, on the column study was demonstrated that a residual concentration of arsenic lower than the maximum limit allowed by the World Health Organization of 10 μg/L can be reached. Compared with other iron loaded adsorbent material mentioned in the literature the proposed one developed a highest adsorption capacity, it can be used with success for arsenic removal from waters containing trace concentrations, the most often situation for drinking waters.
